# SPERMIDINE TOXICITY IN MITOCHONDRIAL DNA-DEFICIENT SACCHAROMYCES CEREVISIAE

**DOI:** 10.1093/geroni/igac059.1740

**Published:** 2022-12-20

**Authors:** Wei-Hsuan Su, Omar Ocegueda, Catherine Choi, Jessica Smith, Kelsey Lee, Yihan Wan, Jacqueline Yao, Sam Schriner

**Affiliations:** University of California, Irvine, Irvine, California, United States; University of California, Irvine, Irvine, California, United States; University of California, Irvine, Irvine, California, United States; University of California, Irvine, Irvine, California, United States; University of California, Irvine, Irvine, California, United States; University of California, Irvine, Irvine, California, United States; University of California, Irvine, Irvine, California, United States; University of California, Irvine, Irvine, California, United States

## Abstract

Mitochondrial dysfunction is thought to play a significant role in aging and in manyhuman diseases. Over the last 20 years or so, a number of drugs have been found toextend lifespan in model organisms. Using ethidium bromide to deplete the yeastSaccharomyces cerevisiae of its mitochondrial DNA (mtDNA), we evaluated thedependence on functional mitochondrial in the action of five of these lifespan-extending compounds; dinitrophenol, metformin, rapamycin, resveratrol, andspermidine. None of them extended lifespan in mtDNA-deficient cells, demonstratinga requirement for functional mitochondria in their action. However, we found thatspermidine significantly shortened lifespan in these cells, decreasing the medianlifespan from 6 days to 4 days. Despite this, spermidine, nor any of the othercompounds tested, had any effect of growth rates in mtDNA-deficient cells.Spermidine is thought to extend lifespan through the induction of autophagy. Wepredict that spermidine shortened lifespan in mtDNA-deficient cells through anincreased need for ATP, for which these cells were not able to provide. Given thatmitochondrial dysfunction might be a common feature of aging and disease, ourresults suggest that if spermidine were used as an anti-aging treatment in humans, itmay be harmful.

